# Socioeconomic Inequalities in Health among Armenian Adolescents

**DOI:** 10.3390/ijerph17114055

**Published:** 2020-06-06

**Authors:** Armen A. Torchyan, Hans Bosma

**Affiliations:** 1 Department of Family and Community Medicine, College of Medicine and King Khalid University Hospital, King Saud University, P.O. Box 7805, Riyadh 11472, Saudi Arabia; 2 Department of Social Medicine, Care and Public Health Research Institute (CAPHRI), Faculty of Health, Medicine and Life Sciences, Maastricht University, P.O. Box 616, 6200 MD Maastricht, The Netherlands; hans.bosma@maastrichtuniversity.nl

**Keywords:** adolescent, Armenia, equalization, HBSC, health status, material well-being, popularity, psychosocial well-being, psychosomatic symptoms, socioeconomic status

## Abstract

We aimed to study the hypothesis of socioeconomic equalization in health among Armenian adolescents participating in the Health Behavior in School-Aged Children 2013/14 survey. Classes corresponding to the ages 11, 13, and 15 were selected using a clustered sampling design. Multiple logistic regression analyses were used. In a nationally representative sample of 3679 students, adolescents with a low family socioeconomic position (SEP) had greater odds of reporting less than good health (odds ratio (OR) = 2.81, 95% CI = 2.25–3.51), low psychosocial well-being (OR = 1.94, 95% CI = 1.44–2.61), or psychosomatic symptoms (OR = 1.29, 95% CI = 1.07–1.56). Low levels of material well-being were associated with a higher likelihood of reporting less than good health (OR = 1.32, 95% CI = 1.06–1.65) or low psychosocial well-being (OR = 1.27, 95% CI = 1.04–1.54). The presence of both risk factors had a synergistic effect on having low psychosocial well-being (*P*-interaction = 0.031). Refuting the equalization hypothesis, our results indicate that low SEP might be strongly related to adolescent health in middle-income countries such as Armenia. Low material well-being also proved important, and, for further research, we hypothesized an association via decreased peer social status and compromised popularity.

## 1. Introduction

Adolescence is a critical developmental period. Substantial physical, emotional, behavioral, and social developmental changes occur during this life period. These can not only affect adolescents’ current well-being, but also impact their health outcomes in later life [1]. Adolescence is a time when most mental disorders are starting to develop and to have negative consequences in terms of poor academic achievement and risky health behaviors, such as substance abuse, violence, and poor sexual health [2].

Many of the factors influencing adolescent health are associated with social determinants, which include socioeconomic status (SES), family and school environment, and relationships with peers. For example, socioeconomically disadvantaged adolescents experience higher rates of psychosomatic symptoms, obesity, and injuries, and lower levels of life satisfaction [3]. Adolescents living in better-off families have a healthier lifestyle, lower levels of risky health behavior, and better mental health and academic performance [4].

The existing literature shows that the influence of SES on adolescent health varies considerably between and within countries, with some studies showing no significant difference in specific health outcomes among adolescents [3,5,6]. Based on findings from Scotland, West [7,8] proposed the equalization in health hypothesis, suggesting that the school environment, peers, and youth culture can substantially attenuate the relationship between SES and health measures and can even lead to relative health equality among adolescents, especially for health outcomes related to physical and mental symptoms. Their hypothesis has been tested in subsequent studies, mostly on high-income countries, with varying results depending on the country, SES, and the health outcome measures that have been used [9,10,11,12]. The equalization hypothesis does not exclude the possibility that there might be other than socioeconomic, health-relevant hierarchies, such as being popular, powerful, respected, attractive, or a trouble-maker, as well as academic or sports performance [13].

Armenia is a post-Soviet middle-income country with approximately a third of the population living in poverty and 72.2% experiencing deprivation in at least one dimension [14]. While some studies show a strong positive relationship between SES and health in the adult population [15], no studies have explored the influence of SES on adolescents’ health in Armenia. Therefore, in this paper, we aimed to study the hypothesis of equalization in health among a nationally representative sample of 3679 Armenian adolescents participating in the Health Behavior in School-Aged Children (HBSC) 2013/14 study by examining the relationship between two measures of SES (family socioeconomic position and material well-being) and three different dimensions of health outcomes (perceived health status, psychosocial well-being, and psychosomatic symptoms).

## 2. Materials and Methods

Data for this study was obtained from the HBSC survey conducted during the years 2013–2014 using a standard methodology [16]. The HBSC 2013/14 is a WHO collaborative cross-national study of the health and health behaviors of schoolchildren aged 11, 13, and 15 years. An international standard anonymous questionnaire was self-administered in schools to collect the data. Appropriate ethical considerations were made in all countries [5].

Adolescents in Armenia were selected using a clustered sampling design. In the first stage, the schools were selected using probability proportional to size (PPS) sampling. Then, the class groups within schools were randomly selected. Only classes corresponding to the 11, 13, and 15 age groups were included. A nationally representative sample was drawn [17].

### 2.1. Outcome Measures

Perceived health status was measured by self-rated health, asking the respondents, “Would you say your health is…?” with the response options ordered as “excellent, good, fair, or poor”. The latter two options were combined to indicate less than good health (15% reported less than good health). Psychosocial well-being was assessed with an adapted version of the Cantril Ladder. Adolescents were shown a picture of a ladder, where the bottom “0” corresponded to the worst possible life for them and the top “10” to the best possible life, and were asked, “In general, where on the ladder do you feel you stand at the moment” [16]. The results were categorized into low (0–8) and high (9–10) levels of psychosocial well-being (35% reported low levels). Similar cut-off points were reported in other studies [18]. Adolescents were considered to have psychosomatic symptoms if they reported two or more subjective health complaints more than once a week in the past six months (35% reported more than one symptom). Subjective complaints included the following symptoms: headache, stomachache, backache, feeling low, irritability or bad temper, feeling nervous, difficulty in getting to sleep, and feeling dizzy [16].

### 2.2. SES Measures

Family socioeconomic position (SEP) was assessed based on adolescents’ responses to the question, “How well off do you think your family is?”. The family SEP was considered to be high if the response was “quite well off” or “very well off”. The response options “average”, “not very well off”, and “not at all well off” were combined into the low family SEP category [16]. Material well-being was measured using the HBSC Family Affluence Scale (FAS), comprising four items: car (3 categories) and computer ownership (4), own bedroom (2), and holidays abroad (4) [16]. The FAS score (ranging from 4 (least affluent) to 13 (most affluent)) was calculated by adding the student responses for each item. Then, the FAS score was divided into low (from 4 to 7) and high (from 8 to 13) categories, based on the median value.

### 2.3. Statistical Analysis

Descriptive statistics examined the distributions and patterns of health outcome variables and sociodemographic characteristics. Simple and multiple logistic regression analyses were used to test the associations between covariates of interest and health outcomes. Three separate regression models were estimated for each of the outcomes. Multivariate models were controlled for age and sex. All the covariates, including age and sex, were checked for two-way interactions (with both SES measures) by separately adding product terms to the model. Pearson’s correlation coefficient (r) was used to calculate the correlation between family SEP and material well-being. A study of the variance inflation factors (VIFs) revealed no multicollinearity problems in the data. The analyses were adjusted for cluster sampling using a complex samples module. A total of 3679 participants were included in the analyses, but the number of participants varied between analyses depending upon the variables included in the models (see Table 1, Table 2, Table 3, Table 4 and Table 5). The statistical analyses were performed using IBM SPSS Statistics for Windows, Version 21.0 (IBM Corp., Armonk, NY, USA).

## 3. Results

During the study period, 3679 students participated in the survey. More than a quarter (29.3%) of students were from low-SEP families, and 51.5% of families had relatively low levels of material well-being. Approximately 15.4% of students reported less than good health, 35.0% had reduced psychosocial well-being, and 35.4% experienced psychosomatic symptoms during the past six months. Descriptive statistics on all variables are presented in Table 1.

In univariate analyses (Table 2 and Table 3), older adolescents had worse psychosocial well-being and more psychosomatic symptoms. Girls were more likely to report less than good health and psychosomatic symptoms. Both low family SEP and low material well-being were statistically significantly associated with worse health outcomes. Although the odds ratio was in a similar direction (i.e., >1.00), there was no statistically significant relationship between material well-being and psychosomatic symptoms.

In multivariate analyses (Table 4), adolescents with low family SEP had almost three times higher odds of reporting less than good health (odds ratio (OR) = 2.81, 95% confidence interval (CI) = 2.25–3.51), 1.94 times greater odds of having low psychosocial well-being (OR = 1.94, 95% CI = 1.44–2.61), and a 1.29-fold increase in the odds of experiencing psychosomatic symptoms (OR = 1.29, 95% CI = 1.07–1.56), compared to respondents with high family SEP. Low levels of material well-being were associated with a higher likelihood of reporting less than good health (OR = 1.32, 95% CI = 1.06–1.65), or low psychosocial well-being (OR = 1.27, 95% CI = 1.04–1.54), but not psychosomatic symptoms (*p* = 0.569), compared to families with high material well-being. Having a family with both low SEP and low material well-being increased the risk of low psychosocial well-being by almost four times (OR = 3.73, 95% CI = 3.00–4.63; *P*-interaction = 0.031), compared to their counterparts with both high family SEP and high material well-being (Table 5). The correlation between family SEP and material well-being was weak in our study population (Pearson’s r = 0.265; *p* < 0.001).

## 4. Discussion

In this nationally representative study, we did not find evidence for equalization in health among Armenian adolescents. The findings indicate that both family SEP and material well-being had a substantial influence on all three health outcomes, except for the relationship between material well-being and psychosomatic symptoms, which was not statistically significant, but for which the odds ratio of poor health was still heightened for adolescents living in poor material circumstances. The presence of both risk factors had a synergistic effect on reporting low psychosocial well-being.

According to West et al. [8], equalization can potentially occur in psychosocial health outcomes that are sensitive to influences from social factors related to youth culture, school environment, and relationships with peers. In particular, they suggested that those health outcomes should mostly include psychosocial malaise, physical symptoms, and to some extent, self-rated health. On the contrary, they hypothesized that the relationship between parental SES and long-standing illnesses or conditions should persist into adolescence, regardless of the influences mentioned above. However, the current findings relating to (bio-)psychosocial health outcomes show no evidence for equalization in Armenian youth, except maybe for the relationship between material well-being and psychosomatic symptoms.

We cannot exclude the possibility that low SES is better able to express its negative consequences in the middle-income Armenian situation, which is characterized by comparatively high poverty rates (29.4%) and an average monthly income per adult equivalent of around USD 118.2 [14]. Hence, adolescents growing up in Armenian low-income families are more likely to live in disadvantageous households, have parents with poor mental and emotional well-being, be poorly supervised, have negative peer influence, experience interpersonal violence, engage in risky health behaviors, and receive a poor-quality education [19,20]. The cumulative effect of those factors, in addition to income-related health differences in very early childhood, can substantially increase the risk of adverse health outcomes in Armenian adolescents.

In our study, low material well-being was associated with a higher likelihood of reporting less than good health and low psychosocial well-being, even when controlling for family SEP. Moreover, when adding an interaction term to the model, low material well-being increased the negative effect of low family SEP on adolescents’ psychosocial well-being. Previous studies showed that adolescents with low material well-being can be less popular among their peers, i.e., have lower peer social status, which can provide a possible explanation of our findings [21,22,23]. The social rank theory of depression suggests that when people experience being of lower status compared to others, feelings of defeat and entrapment can arise, which have been found to be strongly associated with depression [24,25]. Recent studies among adolescents confirmed the relationship between low peer status and depression proposed by the social rank theory [26,27,28].

The SES factors mentioned above are essential not only for the current health and well-being of adolescents but also for their future educational attainment. Education is one of the powerful determinants of health and will eventually determine their occupational and income positions during adulthood. The relationship between low SES and poor academic achievement has been well established. The economic, cultural, and social capital of parents have been proposed as possible pathways for that relationship [29]. Furthermore, low peer social status, as discussed above, can also prevent adolescents from pursuing higher education through the mechanisms proposed by the social rank theory, such as depression and loss of motivation [24,25]. Lower educational levels among the adolescents themselves have been found to affect adolescents’ mental health [10], which potentially creates a vicious cycle of low educational attainment and poor health. This further highlights the importance of our findings, not only for the health status of Armenian adolescents but also for their school careers and the consequences of both for widening socioeconomic health inequalities into adulthood.

The present study has several strengths. The standardized methodology of the HBSC studies allowed us to use high-quality data from a nationally representative survey to test the equalization hypothesis in health among Armenian adolescents. A relatively large sample size provided us with sufficient statistical power to investigate in detail the relationship between different health outcomes and SES measures, including interactions. Having multiple health outcomes improved our understanding of the equalization hypothesis in Armenia. Simultaneously controlling for family SEP and material well-being in the models allowed us to study different aspects of SES.

Our study is not without limitations. The cross-sectional design of the HBSC study did not allow us to establish causal relationships between variables. Self-reported data could have potentially caused biased results, i.e., adolescents with more psychological problems could have reported lower SES. Also, some of the respondents might have interpreted the questions on psychosocial well-being in material terms. Unfortunately, in our study, we had no information on some potential confounders, such as ethnicity and parental divorce. However, these might have minimal impact on our results, since Armenia is a predominantly monoethnic country (98.1%) with a low divorce rate (1.2 per 1000) [30]. The proportion of missing values for psychosomatic symptoms was high (21.9%), with higher prevalence in younger age groups and boys. Nevertheless, it was similar for high and low categories of family SEP and material well-being, and it is thus unlikely that the results were very much affected by missing values. Other variables had fewer missing values (up to 8.6%), with a higher prevalence of missing values in boys but without a regular pattern in other variables.

## 5. Conclusions

In conclusion, refuting the equalization hypothesis, our results indicate that low SEP might be strongly related to adolescent health in middle-income countries, such as Armenia. Low material well-being proved important too, independently or synergistically, which might have significant implications for designing adolescent health programs in middle-income countries. For further research, we hypothesized an association between low material well-being and adolescent health via decreased peer social status and compromised popularity. More research is needed to explore the possible pathways for the relationship between SES and adolescent health in Armenia.

## Figures and Tables

**Table 1:** Characteristics of adolescents (*n* = 3679 participants).

Characteristics	*n*	% ^a^
Age in years		
11	1471	40.0
13	1163	31.6
15	1044	28.4
Missing	1	<0.1
Sex		
Boys	1759	47.8
Girls	1920	52.2
Missing	0	0.0
Family SEP		
High	2425	70.7
Low	1006	29.3
Missing	248	6.7
Material well-being		
High	1631	48.5
Low	1732	51.5
Missing	316	8.6
Less than good health		
Yes	544	15.4
No	2987	84.6
Missing	148	4.0
Low psychosocial well-being		
Yes	1190	35.0
No	2208	65.0
Missing	281	7.6
Psychosomatic symptoms		
Yes	1018	35.4
No	1854	64.6
Missing	807	21.9

SEP, socioeconomic position. ^a^ Participants with missing data were excluded from the calculation of percentages.

**Table 2:** Frequencies (percentages ^a^) of health outcomes by different demographic and socioeconomic characteristics.

Factors	Less than Good Health			Low Psychosocial Well-Being			Psychosomatic Symptoms		
	Yes	No	Missing	Yes	No	Missing	Yes	No	Missing
Age in years ^b^									
11	215 (15.3)	1189 (84.7)	67 (4.5)	351 (25.7)	1015 (74.3)	105 (7.1)	325 (31.1)	720 (68.9)	426 (30.0)
13	162 (14.3)	972 (85.7)	29 (2.5)	371 (34.2)	714 (65.8)	78 (6.7)	337 (35.4)	614 (64.6)	212 (18.2)
15	167 (16.8)	825 (83.2)	52 (5.0)	468 (49.5)	478 (50.5)	98 (9.4)	356 (40.7)	519 (59.3)	169 (16.2)
Sex									
Boys	220 (13.3)	1429 (86.7)	110 (6.3)	536 (33.9)	1045 (66.1)	178 (10.1)	395 (29.8)	929 (70.2)	435 (24.7)
Girls	324 (17.2)	1558 (82.8)	38 (2.0)	654 (36.0)	1163 (64.0)	103 (5.4)	623 (40.2)	925 (59.8)	372 (19.4)
Family SEP ^c^									
High	254 (10.7)	2122 (89.3)	49 (2.0)	631 (27.5)	1662 (72.5)	132 (5.4)	645 (33.5)	1281 (66.5)	499 (20.6)
Low	261 (26.6)	721 (73.4)	24 (2.4)	513 (53.4)	448 (46.6)	45 (4.5)	328 (40.0)	493 (60.0)	185 (18.4)
Material well-being ^d^									
High	188 (11.8)	1407 (88.2)	36 (2.2)	437 (28.2)	1112 (71.8)	82 (5.0)	450 (33.8)	883 (66.2)	298 (18.3)
Low	313 (18.5)	1381 (81.5)	38 (2.2)	684 (41.6)	960 (58.4)	88 (5.1)	510 (37.2)	862 (62.8)	360 (20.8)

SEP, socioeconomic position. ^a^ Participants with missing data were excluded from the calculation of percentages; ^b^ Data were missing for 1 participant; ^c^ Data were missing for 248 participants; ^d^ Data were missing for 316 participants.

**Table 3:** Odds ratios (95% confidence interval) of poor health outcomes by different demographic and socioeconomic characteristics.

Factors	Less than Good Health	Low Psychosocial Well-Being	Psychosomatic Symptoms
Age in years			
11	1.00	1.00	1.00
13	0.92 (0.69–1.22)	**1.50 (1.07–2.11)**	1.22 (0.94–1.57)
15	1.12 (0.85–1.48)	**2.83 (2.05–3.92)**	**1.52 (1.21–1.90)**
Sex			
Boys	1.00	1.00	1.00
Girls	**1.35 (1.11–1.65)**	1.10 (0.94–1.28)	**1.58 (1.33–1.89)**
Family SEP			
High	1.00	1.00	1.00
Low	**3.02 (2.43–3.77)**	**3.02 (2.53–3.60)**	**1.32 (1.10–1.59)**
Material well-being			
High	1.00	1.00	1.00
Low	**1.70 (1.39–2.08)**	**1.81 (1.56–2.11)**	1.16 (0.99–1.37)

SEP, socioeconomic position; bold values denote statistical significance (*p* < 0.05). Missing data for all variables are provided in Table 2.

**Table 4:** Odds ratios (95% confidence interval) of poor health outcomes by family socioeconomic position and material well-being, adjusted for age and sex, and simultaneously controlling for both SES measures.

Factors	Less than Good Health		Low Psychosocial Well-Being		Psychosomatic Symptoms	
	OR (95% CI) ^a^	*p*-Value	OR (95% CI) ^b^	*p*-Value	OR (95% CI) ^c^	*p*-Value
Family SEP						
High	1.00		1.00		1.00	
Low	**2.81 (2.25–3.51)**	**<0.001**	**1.94 (1.44–2.61)**	**<0.001**	**1.29 (1.07–1.56)**	**0.008**
Material well-being						
High	1.00		1.00		1.00	
Low	**1.32 (1.06–1.65)**	**0.014**	**1.27 (1.04–1.54)**	**0.019**	1.05 (0.89–1.25)	0.569

The model for low psychosocial well-being includes a product term for family SEP and material well-being. Bold values denote statistical significance (*p* < 0.05). ^a^ Data were missing for 427 participants (*n* = 3252 participants); ^b^ Data were missing for 515 participants (*n* = 3164 participants); ^c^ Data were missing for 996 participants (*n* = 2683 participants). Abbrevations: OR, odds ratio; SES, socioeconomic status.

**Table 5:** Odds ratios (95% confidence interval) of low psychosocial well-being by the interaction of family socioeconomic position and material well-being, adjusted for age and sex.

Material Well-Being			
Family SEP	High OR (95% CI)	Low OR (95% CI)	Low vs. High OR (95% CI)
High	1.00	**1.27 (1.04–1.54)** ***p* = 0.019**	**1.27 (1.04–1.54)** ***p* = 0.019**
Low	**1.94 (1.44–2.61)** ***p* < 0.001**	**3.73 (3.00–4.63)** ***p* < 0.001**	**1.93 (1.40–2.65)** ***p* < 0.001**
Low vs. High	**1.94 (1.44–2.61)** ***p* < 0.001**	**2.95 (2.32–3.75)** ***p* < 0.001**	

Measure of interaction on multiplicative scale: ratio of ORs (95% CI) = 1.52 (1.04–2.23); *p* = 0.031. Bold values denote statistical significance (*p* < 0.05).
